# *APOE-ε4* modulates the association between regional amyloid deposition and cognitive performance in cognitively unimpaired middle-aged individuals

**DOI:** 10.1186/s13550-023-00967-6

**Published:** 2023-03-01

**Authors:** Anna Brugulat-Serrat, Gonzalo Sánchez-Benavides, Raffaele Cacciaglia, Gemma Salvadó, Mahnaz Shekari, Lyduine E. Collij, Christopher Buckley, Bart N. M. van Berckel, Andrés Perissinotti, Aida Niñerola-Baizán, Marta Milà-Alomà, Natàlia Vilor-Tejedor, Grégory Operto, Carles Falcon, Oriol Grau-Rivera, Eider M. Arenaza-Urquijo, Carolina Minguillón, Karine Fauria, José Luis Molinuevo, Marc Suárez-Calvet, Juan Domingo Gispert, the ALFA Study, Alba Cañas, Lidia Canals, Laura Iglesias, Paula Marne, Annabella Beteta, Carme Deulofeu, Maria Emilio, Irene Cumplido, Ruth Domínguez, Sherezade Fuentes, Laura Hernández, Marc Vilanova, Lluís Solsona, Gema Huesa, Jordi Huguet, Tania Menchón, Albina Polo, Sandra Pradas, Aleix Sala-Vila, Anna Soteras, Laura Stankeviciute, Müge Akinci, Eleni Palpatzis, Patricia Genius, Blanca Rodríguez, Marina García, Paula Ortiz-Romero

**Affiliations:** 1 Barcelonaβeta Brain Research Center (BBRC), Pasqual Maragall Foundation, Wellington 30, 08005 Barcelona, Spain; 2 IMIM (Hospital del Mar Medical Research Institute), Barcelona, Spain; 3 Centro de Investigación Biomédica en Red de Fragilidad Y Envejecimiento Saludable (CIBERFES), Instituto de Salud Carlos III, Madrid, Spain; 4 Global Brain Health Institute, San Francisco, CA USA; 5 Department of Clinical Sciences, Clinical Memory Research Unit, Lund University, Lund, Sweden; 6 Universitat Pompeu Fabra, Barcelona, Spain; 7 Department of Radiology and Nuclear Medicine, Amsterdam UMC, Vrije Universiteit Amsterdam, De Boelelaan, Amsterdam, The Netherlands; 8 Center for Medical Image Computing, and Queen Square Institute of Neurology, UCL, London, UK; 9 Nuclear Medicine Department, Hospital Clínic, Barcelona, Spain; 10 Biomedical Research Networking Center of Bioengineering, Biomaterials and Nanomedicine (CIBER-BBN), Instituto de Salud Carlos III, Madrid, Spain; 11 Centre for Genomic Regulation (CRG), The Barcelona Institute for Science and Technology, Barcelona, Spain; 12 Neurologia Department, Hospital del Mar, Barcelona, Spain; 13 H. Lundbeck A/S, Copenhagen, Denmark

**Keywords:** Alzheimer’s disease, Amyloid PET, Visual read, Memory, Executive function, *APOE-ε4*

## Abstract

**Purpose:**

To determine whether the *APOE-ε4* allele modulates the relationship between regional β-amyloid (Aβ) accumulation and cognitive change in middle-aged cognitively unimpaired (CU) participants.

**Methods:**

The 352 CU participants (mean aged 61.1 [4.7] years) included completed two cognitive assessments (average interval 3.34 years), underwent [^18^F]flutemetamol Aβ positron emission tomography (PET), T1w magnetic resonance imaging (MRI), as well as *APOE* genotyping. Global and regional Aβ PET positivity was assessed across five regions-of-interest by visual reading (VR) and regional Centiloids. Linear regression models were developed to examine the interaction between regional and global Aβ PET positivity and *APOE-ε4* status on longitudinal cognitive change assessed with the Preclinical Alzheimer’s Cognitive Composite (PACC), episodic memory, and executive function, after controlling for age, sex, education, cognitive baseline scores, and hippocampal volume.

**Results:**

In total, 57 participants (16.2%) were VR+ of whom 41 (71.9%) were *APOE-ε4* carriers. No significant APOE-ε4*global Aβ PET interactions were associated with cognitive change for any cognitive test. However, *APOE-ε4* carriers who were VR+ in temporal areas (*n* = 19 [9.81%], *p* = 0.04) and in the striatum (*n* = 8 [4.14%], *p* = 0.01) exhibited a higher decline in the PACC. The temporal areas findings were replicated when regional PET positivity was determined with Centiloid values. Regionally, VR+ in the striatum was associated with higher memory decline. As for executive function, interactions between *APOE-ε4* and regional VR+ were found in temporal and parietal regions, and in the striatum.

**Conclusion:**

CU *APOE-ε4* carriers with a positive Aβ PET VR in regions known to accumulate amyloid at later stages of the Alzheimer’s disease (AD) continuum exhibited a steeper cognitive decline. This work supports the contention that regional VR of Aβ PET might convey prognostic information about future cognitive decline in individuals at higher risk of developing AD.

*ClinicalTrials.gov Identifier*: NCT02485730. Registered 20 June 2015 https://clinicaltrials.gov/ct2/show/NCT02485730 and ClinicalTrials.gov Identifier:NCT02685969. Registered 19 February 2016 https://clinicaltrials.gov/ct2/show/NCT02685969.

**Supplementary Information:**

The online version contains supplementary material available at 10.1186/s13550-023-00967-6.

## Introduction

Alzheimer’s disease (AD) comprises a long asymptomatic preclinical stage characterized by pathophysiological changes that start decades before overt clinical manifestations [1]. Abnormal brain accumulation of β-amyloid (Aβ) is thought to be among the earliest detectable events occurring along the AD continuum, followed by tau aggregation and cerebral atrophy [1]. PET imaging allows the detection of Aβ plaques across the brain in vivo. In clinical settings, Aβ PET scans are typically assessed visually and categorized as negative or positive. To this end, scans are assessed in several brain regions and categorized as positive if significant Aβ is detected in at least one culprit brain region. According to an investigation of this type, Aβ accumulation can be detected in cognitively unimpaired (CU) individuals decades before the onset of clinical symptoms [2]. In addition, there is strong evidence suggesting that global Aβ positivity in CU individuals is associated with future cognitive decline [3–5].

Even though the regional pattern of Aβ positivity is usually not taken into consideration in clinical routine, there is evidence pointing that the consideration of this regional pattern is of prognostic value [6], as Aβ accumulation typically follows a defined spatial–temporal pattern progression across the AD continuum [7]. Cortical regions, such as the precuneus, insular, cingulate, and orbital cortices, generally show Aβ deposition earlier than temporal or striatal regions [7]. In line with this, recent literature shows that changes in regional Aβ, as measured by PET quantification, can predict episodic memory decline in CU individuals, particularly in the precuneus [8, 9], posterior cingulate cortex, and lateral parietal cortices [9]. Therefore, regional Aβ PET measures may be better suited for predicting cognitive decline than global positivity as defined when any region is read as positive. Specifically, regional Aβ PET analyses could potentially be useful for intervention trials, insofar as Aβ positivity in key brain regions might identify those CU individuals who are at greater risk for developing AD [10, 11].

The *APOE-ε4* allele represents the major genetic factor for non-autosomal inherited AD, and it is associated with an earlier and higher cerebral Aβ deposition [12–14] which is proportional to the number of *ε4* alleles [12]. Carrying this allele is also associated with a greater risk of AD dementia, younger age of symptoms onset, and faster cognitive decline [15]. However, the impact of the *APOE-ε4* allele on cognition in late-/middle-aged CU individuals remains unclear. Previous studies reported a faster cognitive decline in *ε4* carriers than in non-carriers [16, 17], especially when individuals are Aβ positive [18–20], although another study with a similar design did not confirm such longitudinal association [21]. Similarly, cross-sectional studies reported worse cognitive performance in CU *ε4* carriers compared to non-carriers [22, 23]. The relationship between regional Aβ accumulation and *APOE-ε4* status associated with cognition in CU has been scantily explored. In a previous study including 408 CU, Kantarci et al. [18] showed that *APOE-ε4* carriers displaying Aβ accumulation in frontal, temporal, and parietal lobes presented worse global cognitive performance.

In the present study, we aimed to determine whether the presence of the *APOE-ε4* allele modifies the association between global and regional Aβ PET visual reads (VR) and cognitive decline in middle-aged CU individuals. We hypothesized that (1) the *APOE-ε4* allele modulates the relationship between regional Aβ burden and cognitive changes in the early asymptomatic stages of the AD continuum*,* and (2) regional Aβ positivity is predictive of cognitive decline in CU individuals at higher risk of developing AD. We primarily assessed cognitive decline by means of the Preclinical Alzheimer Cognitive Composite (PACC), wherein we separately analysed scores for episodic memory and executive function. We further examined these relationships using regional positivity determinations based on standardized update value ratio (Centiloids) as a quantitative measurement of Aβ deposition and explored whether the studied associations could be related to hippocampal volume as a measure of neurodegeneration.

## Methods

### Subjects

**Fig. 1:**
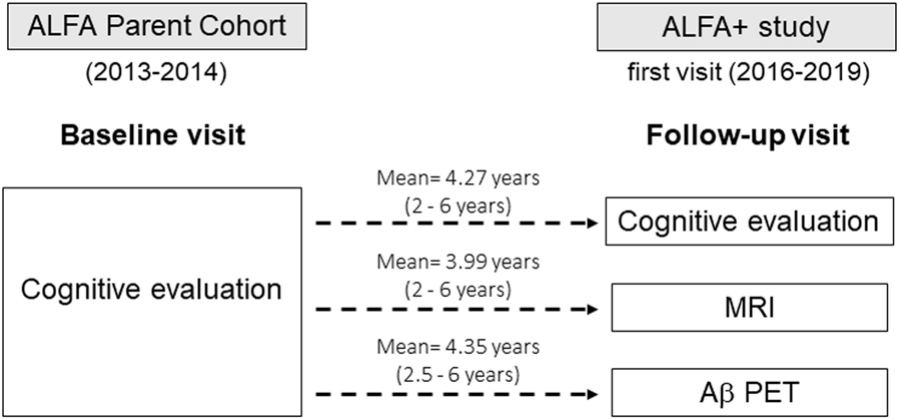
Schematic representation of the time between baseline visit and follow-up procedures

The ALFA+ cohort is a nested longitudinal study of the ALFA (for ALzheimer and Families) parent cohort [24]. The ALFA parent cohort was established as a research platform to understand the early pathophysiological alterations in preclinical AD and is composed of 2743 CU individuals (between 45 and 75 years) and enriched for family history of AD and genetic risk factors for AD (namely presence of the *APOE-ɛ4* allele). In the present study, we included the first consecutive 352 participants of the ALFA+ cohort with two cognitive assessments (the first in the context of the ALFA parent cohort baseline visit [2013–2014] and the second from the baseline visit of the ALFA+ study [2016–2019]) who also had available [^18^F]flutemetamol Aβ PET and T1w MRI in the follow-up, as well as *APOE* genotyping (Fig. 1).

### APOE genotyping

Total DNA was obtained from cellular blood fraction by proteinase K digestion followed by alcohol precipitation. Samples were genotyped for two single nucleotide polymorphisms (SNPs), *rs429358* and *rs7412*, to define the *APOE-ε2*, *ε3*, and *ε4* alleles. In this study, participants were classified as *ε4* carriers (one or two alleles) or *ε4* non-carriers.

### Cognitive measures

The main cognitive outcome was the PACC that was computed including the Total Paired Recall (TPR) and Total Delayed Free Recall scores of the Memory Binding Test [25], the Coding subtest of the Wechsler Adult Intelligence Scale-Fourth Edition (WAIS-IV), and semantic fluency, as defined in previous works [26, 27].

In addition to the PACC, episodic memory and executive function measures were also analysed. The TPR score was used as a measure of verbal episodic memory. Previous studies have established its ability to discriminate individuals with amnestic cognitive impairment from normal elderly individuals [28, 29]. Executive functions were assessed with five WAIS-IV subtests: the Digit Span; Coding subtest; and Matrix Reasoning and Visual Puzzles. Cognitive change for each test was computed as follows, so that negative values reflect worse performance in the follow-up visit:

$$Cognitive change = \frac{{follow{ - }up cognitive score - baseline cognitive score}}{{\text{time (years)}}}$$

### Aβ PET: acquisition and analysis

Participants received a bolus injection of 185 MBq (range 104.25–218.3 MBq, mean ± SD 191.75 ± 14.04 MBq) of [18F]flutemetamol. Scans were acquired 90 min post-injection on a Siemens Biograph mCT. PET data were reconstructed into 4 frames of 5 min after correcting for radioactive decay, dead time, attenuation, and scatter. Global Standard Uptake Values ratios (SUVRs) were calculated in MNI space using the target region provided in the GAAIN website (www.gaain.org) using the whole cerebellum as reference region and converted to Centiloids units using a previously validated equation [30]. Regional SUVRs (reference: whole cerebellum) were extracted on the frontal, precuneus/posterior cingulate (PCPCC), lateral–parietal, and lateral temporal cortices, as well as in the striatum using the Desikan Killiany atlas [31] and converted to regional Centiloids units using the global conversion equation [32].

### Visual assessment of PET scans

All 352 scans were read by one experienced reader (LEC). Scans were visually rated as positive (VR+) or negative (VR−) using standard clinical criteria as specified in the Summary of Product Characteristics (SmPC; https://www.ema.europa.eu/en/documents/product-information/vizamyl-epar-product-information_en.pdf) of the tracer. In line with these criteria, 5 regions were assessed: frontal cortex, PCPCC, lateral–parietal, lateral temporal, and striatum. The global classification was also available, with images rated as either positive (unilateral binding in one or more cortical brain regions or striatum) or negative (predominantly white matter uptake). For further details about regional VR rating please refer to [6]. With the aim of replicating the results obtained with the regional VR using a quantitative metric, regional Centiloids values were dichotomized as positive (Centiloid+) or negative (Centiloid-) using region-specific cut-off values of data (*N* = 497) from two cohorts: the ALFA+ cohort and the Dutch Flutemetamol study from the Amsterdam Dementia Cohort (ADC) [33]. These cut-offs were derived to optimize the agreement between regional VR and regional quantification by means of selecting the highest Youden’s index when comparing both measures in each region independently.

### Hippocampal volumes

Scans were obtained with a 3-T Magnetic Resonance scanner (Ingenia CX, Philips, Amsterdam, Netherlands). The MRI protocol included a 3D T1-weighted Turbo Field Echo sequence (voxel size 0.75 × 0.75 × 0.75 mm, TR/TE: 9.90/4.6 ms, fip angle = 8). FreeSurfer version 6.0 was used to segment of the hippocampus from the T1-weighted scans. A bilateral hippocampal volume variable was constructed by summing up the measurements of the left and right hemispheres. TIV-adjusted hippocampal volumes (HVa) were calculated as the residuals of a linear regression using total intracranial volume (TIV) as independent variable. HVa is a well-established measure of neurodegeneration [34–36] and an indicator of AD severity/stage [37, 38].

### Statistical analysis

Participants were categorized according to global VR results as positive (VR+) and negative (VR−). Sociodemographic characteristics and clinical data were compared between groups by means of *t* tests or Chi-squared tests, as appropriate. The correlation between Centiloids among regions was assessed by Spearman’s correlations. Differences in the frequencies of *APOE-ε4* carriership with regional amyloid positivity, both VR and regional Centiloids, were assessed by Chi-square tests. Our main analysis was set up to investigate whether *APOE-ε4* status changed the association between global and regional positivity of Aβ PET and cognitive change. To this end, we constructed a set of independent linear regression models, one for each outcome (global and regional VR and regional Centiloids) and cognitive measure (PACC and measurements of specific cognitive domains). Measurements of change in cognition were set as dependent variables and predictors included *APOE-ε4* status and Aβ PET positivity (A), and their interaction (APOE-ε4*A). Age, education (not centred), sex, and cognitive baseline scores (not centred) were set as covariates:

$$\begin{aligned} Cognitive change & = APOE{ - }\varepsilon 4*A + APOE{ - }\varepsilon 4 + A + age \\ & \quad + education + sex + cognitive baseline score \\ \end{aligned}$$

To explore the possible role of neurodegeneration in these associations, we computed additional linear regression models including HVa as a covariate. Lastly, we investigated whether the number of positive regional regions in both VR and Centiloids has an impact on cognitive change in *APOE-ε4* carriers by constructing a set of independent linear regression models, one for each significant cognitive measure (PACC, MBT TPR, Digit Span Backward, and Coding). We applied a false discovery rate (FDR) multiple comparison correction against all tested interaction *p*-values following the Benjamini–Hochberg procedure [39]. Significance was assumed at the level of nominal *p* < 0.05, but *p*_*FDR*_ values are also provided. Statistical Package for the Social Sciences (SPSS) version 28.0 and R version 3.6.0 were used for statistical analyses.

## Results

### Sample characteristics

Fifty-seven participants (16.2%) were classified as VR+ globally. The VR+ group was significantly older and encompassed a higher proportion of *APOE-ε4* carriers than the VR- one (Table 1). Global Centiloids mean value was 4.47 (SD=18.34). Regarding regional VR+, frontal (*n* = 48, 13.6%), PCPCC (*n* = 45, 12.8%), and temporal (*n* = 29, 8.2%) regions were the most frequently reported, followed by parietal (*n* = 17, 4.8%) and striatum (*n* = 14, 3.9%) (Table 2).

**Table 1:** Participants’ characteristics by global visual read result

	Total	VR−	VR+	*p*
	(*n* = 352)	(*n* = 295, 83.8%)	(*n* = 57, 16.2%)	
Age, y	61.1 (4.7)	60.5 (4.5)	63.9 (4.2)	< 0.001*
Education, y	13.4 (3.5)	13.5 (3.5)	12.9 (3.8)	0.21
Females	216 (61.4)	182 (61.7)	34 (59.6)	0.77
*APOE-ε4* carriers	193 (54.8)	152 (51.5)	41 (71.9)	0.01*
*Annualized cognitive change*				
PACC	0.01 (0.12)	0.01 (0.11)	− 0.01 (0.13)	0.12
Total paired recall	0.20 (0.91)	0.23 (0.90)	0.02 (0.94)	0.08
Visual puzzles	− 0.04 (0.94)	− 0.04 (0.95)	− 0.06 (0.89)	0.72
Matrix reasoning	− 0.04 (1.09)	− 0.04 (1.05)	− 0.03 (1.30)	0.94
Digit span forward	− 0.03 (0.31)	− 0.02 (0.29)	− 0.03 (0.38)	0.95
Digit span backward	− 0.01 (0.31)	0.00 (0.33)	− 0.01 (0.29)	0.99
Digit span sequencing	− 0.01 (0.37)	0.01 (0.35)	− 0.06 (0.46)	0.34
Coding	− 0.17 (2.23)	− 0.05 (2.26)	− 0.75 (1.99)	0.07
Centiloids	4.47 (18.34)	4.67 (18.63)	3.30 (17.04)	0.917
Total hippocampal volume, mm^3^	7500 (700.9)	7512 (693.9)	7437 (739.9)	0.54
Total intracranial volume, cm^3^	1445.8 (175.7)	1447.1 (170.1)	1439.2 (203.9)	0.76

Data are expressed as mean and SD or number of participants and percentage, as appropriate

*PACC* Preclinical Alzheimer’s Cognitive Composite, *TIV* total intracranial volume, *VR* visual read

*p < 0.05

**Table 2:** Distribution of amyloid positivity and *APOE-ε4* status

	Total	*APOE-ε4* status		*p*
		Carriers	Non-carriers	
	(*n* = 352)	(*n* = 193, 54.8%)	(*n* = 159, 47.2%)	
*Visual read*				
Frontal	48 (13.64)	33 (68.75)	15 (31.25)	0.04*
PCPCC	45 (12.78)	32 (71.11)	13 (28.89)	0.02*
Temporal	29 (8.24)	19 (65.51)	10 (34.49)	0.22
Parietal	17 (4.83)	11 (64.70)	6 (35.30)	0.46
Striatum	14 (3.98)	8 (57.14)	6 (42.86)	0.86
*Regional Centiloid*				
Frontal	50 (14.20)	40 (80.0)	10 (20.0)	< 0.001*
PCPCC	49 (13.92)	35 (71.42)	14 (28.57)	0.01*
Temporal	25 (7.10)	17 (68.0)	8 (32.0)	0.17
Parietal	25 (7.10)	17 (68.0)	8 (32.0)	0.17
Striatum	18 (5.11)	12 (66.67)	6 (33.33)	0.30

Data are expressed as number of participants and percentage

*PCPCC* Precuneus/posterior cingulate cortex

*p < 0.05

All Centiloids regions were highly correlated between them (*p* < 0.001) (see Additional file 1: Fig. S1). Regional Centiloids positivity was determined with the following cut-offs: ≥ 17 for global, ≥ 18 for frontal, ≥ 36 for PCPCC, ≥ 23for temporal, ≥ 26 for parietal, and ≥ 60 for striatum. The binary SUVR-based classification showed a very high concordance against regional VR with an overall percentage of agreement of 96.2% in frontal, 91.8% in PCPCC, 86.2% in temporal, 68.0% in parietal, and 77.8% in the striatum. Globally, 53 (15.06%) participants were classified as Centiloid+. Similar to regional VR, frontal (*n* = 50, 14.20%), PCPCC (*n* = 49, 13.9%), temporal (*n* = 25, 7.10%), and parietal (*n* = 25, 7.10%) were the most frequently reported, followed by striatum (*n* = 18, 5.11%) (Table 2). Comparing both classifications, VR+ was significantly more frequently reported than regional Centiloids in both global (*p* < 0.001) and regional (*p* < 0.001) assessments.

### Distribution of amyloid positivity and *APOE-ε4* status

*APOE-ε4* carriers were significantly more likely to be VR+ in frontal (*n* = 33 [68.75%], *p* = 0.04) and PCPCC (*n* = 32, [71.11%], *p* = 0.02) regions than non-carriers. We did not find significant differences between carriers and non-carriers in the distribution of positive/negative VR in temporal (*n* = 19, [65.51%], *p* = 0.22), parietal (*n* = 11, [64.70%], *p* = 0.46), and striatum (*n* = 8, [57.14%], *p* = 0.86) regions (Table 2). When determining regional positivity with Centiloids, we found that the *ε4* carriers group showed a significantly higher number of Centiloids+ individuals in frontal regions (*n* = 40, [80.0], *p* < 0.001) and PCPCC (*n* = 8, [57.1], *p* ≤ 0.01). No significant differences in the distribution in other regions were found (Table 2).

### Association between global VR, *APOE-ε4* status, and cognitive change

**Fig. 2:**
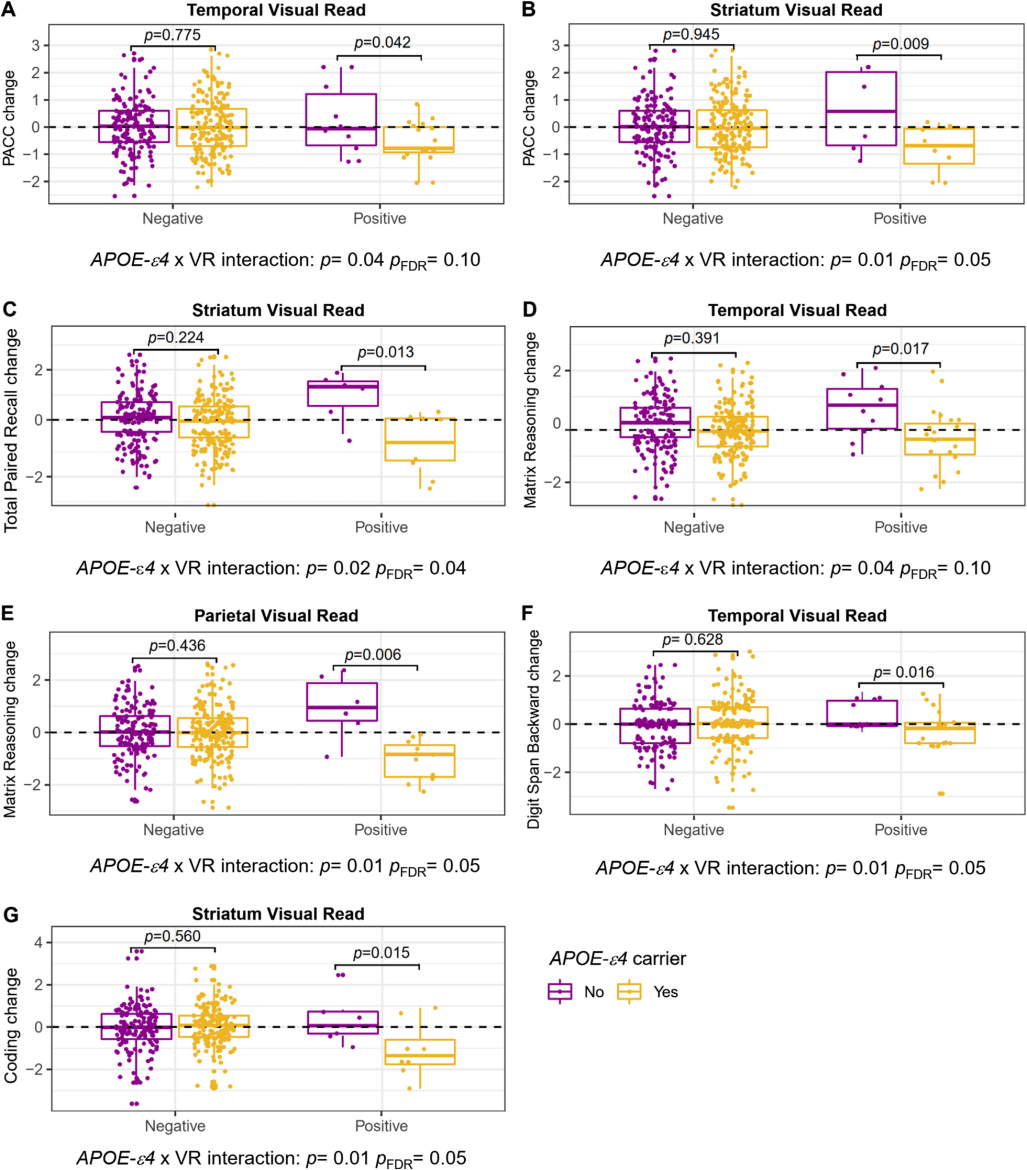
Cognitive change in regional Aβ PET visual read by *APOE-e4* status. Boxplots showing the annualized cognitive change residuals (adjusted by sex, age, education, and baseline score). *P*-values for pairwise comparisons are provided. The dashed line represents stable performance. We computed the *APOE-ε4* x VR interaction term

First, we explored whether the presence of the *APOE-ε4* allele modifies the association between global VR and cognitive decline, as measured with the PACC. *APOE-ε4* status did not interact with global VR+ to determine PACC change (*p* = 0.38, *p*_FDR_ = 0.50), nor did with global Centiloids+ (*p* = 0.86; *p*_FDR_ = 0.86) (Table 3). Next, we investigated whether the *APOE-ε4* allele has an impact on the association between regional Aβ PET positivity and cognitive decline. We found that *APOE-ε4* carriers with VR+ in temporal (*β* = − 0.79, *p* = 0.04, *p*_FDR_ = 0.10) and striatum (*β* = − 1.32, *p* = 0.01, *p*_FDR_ = 0.05) exhibited a significantly worse PACC performance in the follow-up visit (Table 3 and Fig. 2). In the regional Centiloids analyses, the interaction in temporal regions (*β* = − 0.92, *p* = 0.03, *p*_FDR_ = 0.05) was the only that remained significant (Table 3).

**Table 3:** Results of linear regression models examining the interaction between regional Aβ accumulation and APOE*-*ε4 status on cognitive change

	PACC			TPR			Visual Puzzles			Matrix		
	β (SE)	*p*	*p*_FDR_	β (SE)	*p*	*p*_FDR_	β (SE)	*p*	*p*_FDR_	β (SE)	*p*	*p*_FDR_
*APOE-ε4* status × Global												
VR	− 0.26 (0.30)	0.38	0.50	− 0.29 (0.29)	0.30	0.38	− 0.24 (0.28)	0.38	0.52	− 0.41 (0.28)	0.14	0.20
CL	− 0.06 (0.31)	0.86	0.86	− 0.06 (0.29)	0.83	0.94	− 0.38 (0.29)	0.18	0.21	− 0.37 (0.29)	0.20	0.27
*APOE-ε4* status × Frontal												
VR	− 0.20 (0.31)	0.51	0.51	− 0.27 (0.30)	0.35	0.44	− 0.37 (0.30)	0.20	0.24	− 0.44 (0.29)	0.13	0.13
CL	− 0.09 (0.35)	0.81	0.83	− 0.06 (0.33)	0.85	0.92	− 0.51 (0.32)	0.12	0.12	− 0.58 (0.32)	0.08	0.12
*APOE-ε4* status × PCPCC												
VR	− 0.25 (0.32)	0.44	0.51	− 0.14 (0.31)	0.65	0.65	− 0.18 (0.30)	0.65	0.76	− 0.49 (0.30)	0.11	0.13
CL	− 0.17 (0.31)	0.60	0.79	− 0.16 (0.30)	0.59	0.68	− 0.13 (0.30)	0.66	0.75	− 0.41 (0.29)	0.16	0.24
*APOE-ε4* status × Temporal												
VR	− 0.79 (0.38)	0.04*	0.10	− 0.54 (0.36)	0.15	0.25	0.08 (0.36)	0.82	0.82	− 0.72 (0.35)	0.04*	0.10
CL	− 0.92 (0.41)	0.03*	0.05	− 0.71 (0.39)	0.07	0.10	− 0.12 (0.39)	0.77	0.84	− 0.78 (0.34)	0.04*	0.07
*APOE-ε4* status × Parietal												
VR	− 0.83 (0.48)	0.08	0.13	− 0.78 (0.46)	0.09	0.23	− 0.14 (0.46)	0.75	0.86	− 1.16 (0.45)	0.01*	0.05
CL	− 0.49 (0.41)	0.23	0.32	− 0.36 (0.39)	0.35	0.40	− 0.02 (0.39)	0.96	0.96	− 0.77 (0.39)	0.04*	0.07
*APOE-ε4* status × Striatum												
VR	− 1.32 (0.51)	0.01*	0.05	− 1.07 (0.48)	0.02*	0.04*	0.22 (0.48)	0.66	0.77	− 0.86 (0.49)	0.08	0.13
CL	− 0.72 (0.48)	0.13	0.21	− 0.37 (0.46)	0.42	0.49	0.39 (0.45)	0.38	0.51	0.04 (0.45)	0.93	0.93

	DS Forward			DS Backward			DS Sequencing			Coding		
	β (SE)	*p*	*p*_FDR_	β (SE)	*p*	*p*_FDR_	β (SE)	*p*	*p*_FDR_	β (SE)	*p*	*p*_FDR_
*APOE-ε4* status × Global												
VR	0.29 (0.26)	0.27	0.37	− 0.23 (0.27)	0.39	0.68	− 0.02 (0.26)	0.92	0.92	− 0.06 (0.29)	0.85	0.85
CL	0.48 (0.27)	0.07	0.11	− 0.29 (0.29)	0.31	0.41	− 0.05 (0.27)	0.85	0.85	− 0.12 (0.32)	0.70	0.88
*APOE-ε4* status × Frontal												
VR	0.31 (0.27)	0.25	0.31	− 0.01 (0.29)	0.96	0.96	0.02 (0.27)	0.93	0.96	− 0.13 (0.31)	0.68	0.68
CL	0.46 (0.31)	0.13	0.18	− 0.40 (0.32)	0.21	0.34	0.07 (0.30)	0.81	0.81	− 0.16 (0.35)	0.64	0.80
*APOE-ε4* status × PCPCC												
VR	0.35 (0.28)	0.22	0.31	− 0.27 (0.30)	0.37	0.46	0.06 (0.28)	0.82	0.96	− 0.38 (0.33)	0.25	0.31
CL	0.19 (0.28)	0.48	0.55	− 0.26 (0.29)	0.38	0.60	− 0.01 (0.27)	0.97	0.97	− 0.22 (0.32)	0.49	0.65
*APOE-ε4* status × Temporal												
VR	0.19 (0.33)	0.56	0.56	− 0.85 (0.35)	0.01*	0.05	− 0.23 (0.33)	0.49	0.96	− 0.55 (0.38)	0.15	0.31
CL	0.40 (0.36)	0.27	0.34	− 0.66 (0.38)	0.08	0.22	0.09 (0.36)	0.80	0.80	− 0.70 (0.42)	0.09	0.12
*APOE-ε4* status × Parietal												
VR	0.62 (0.43)	0.15	0.32	− 0.60 (0.45)	0.19	0.46	0.02 (0.42)	0.96	0.96	− 0.59 (0.49)	0.25	0.31
CL	0.46 (0.36)	0.20	0.27	− 0.62 (0.38)	0.11	0.26	− 0.15 (0.36)	0.67	0.77	− 0.65 (0.42)	0.12	0.16
*APOE-ε4* status × Striatum												
VR	0.66 (0.45)	0.14	0.32	− 0.45 (0.48)	0.35	0.46	0.03 (0.44)	0.95	0.96	− 1.28 (0.52)	0.01*	0.05
CL	0.24 (0.42)	0.57	0.64	− 0.09 (0.44)	0.83	0.86	0.03 (0.41)	0.07	0.95	− 1.38 (0.48)	0.01*	0.02*

Model: Cognitive change = VR or regional CL x *APOE-ε4* status + regional CL or VR + *APOE-ε4* status + age + sex + education + cognitive baseline score

*CL* Centiloids, *DS* digit span, *PACC* Preclinical Alzheimer Cognitive Composite, *PCPCC* precuneus/posterior cingulate cortex, *TPR* Total Paired Recall, *VR* visual read

*p < 0.05

### Association between regional Aβ accumulation, *APOE-ε4* status, and change in specific cognitive domains

We determined what cognitive domains are associated with specific regional patterns of Aβ positivity (Table 3). *APOE-ε4* status did not interact with global VR+ for any specific tests explored (TPR *p* = 0.30, *p*_FDR_ = 0.38; Visual Puzzles *p* = 0.38 *p*_FDR_ = 0.52; Matrix Reasoning *p* = 0.14, *p*_FDR_ = 0.20; Digit Span Forward *p* = 0.27, *p*_FDR_ = 0.37; Digit Span Backward* p* = 0.39, *p*_FDR_ = 0.68; Digit Span Sequencing *p* = 0.92, *p*_FDR_ = 0.92, and Coding *p* = 0.85, *p*_FDR_ = 0.85). Likewise, no significant effects were detected with global Centiloids+ in any cognitive domain (TPR *p* = 0.83, *p*_FDR_ = 0.94; Visual Puzzles *p* = 0.18 *p*_FDR_ = 0.21; Matrix Reasoning *p* = 0.20, *p*_FDR_ = 0.27; Digit Span Forward *p* = 0.07, *p*_FDR_ = 0.11; Digit Span Backward* p* = 0.31, *p*_FDR_ = 0.41; Digit Span Sequencing *p* = 0.85, *p*_FDR_ = 0.85, and Coding *p* = 0.70, *p*_FDR_ = 0.88).

Regarding regional Aβ positivity, we found an APOE-ε4*VR interaction for memory and executive function domains (Table 3 and Fig. 2). For TPR, *APOE-ε4* carriers with VR+ in the striatum (β = − 1.07, *p* = 0.02, *p*_FDR_ = 0.04) exhibited worse cognitive performance in the follow-up visit. In the Matrix Reasoning test, the significance was shown in temporal (*β* = − 0.72, *p* = 0.04, *p*_FDR_ = 0.10) and parietal (*β* = − 1.16, *p* = 0.01, *p*_FDR_ = 0.05) regions. In addition, we found that *APOE-ε4* carriers with a higher number of positive regions after visual reading exhibited worse Matrix Reasoning performance in the follow-up visit (see Additional file 1: Table S1). For the Digit Span Backward the significance was found in temporal regions (*β* = − 0.85, *p* = 0.01, *p*_FDR_ = 0.05) and for Coding test performance in the striatum (*β* = − 1.28, *p* = 0.01, *p*_FDR_ = 0.05).

In the regional Centiloids analyses (Table 3), Matrix in temporal (*β* = − 0.78, *p* = 0.04, *p*_FDR_ = 0.07) and parietal regions (*β* = − 0.77, *p* = 0.04, *p*_FDR_ = 0.07), and Coding in striatum (*β* = − 1.38, *p* = 0.01, *p*_FDR_ = 0.02) remained significant.

### Effect of hippocampal volume

Adding HVa as covariate the interactions in temporal regions remained significant for both regional VR (*β* = − 0.9, *p* = 0.04, *p*_FDR_ = 0.13) and Centiloids (*β* = − 1.20, *p* = 0.04, *p*_FDR_ = 0.18) for PACC. In addition, the interaction with regional VR (*β* = − 1.13, *p* = 0.04, *p*_FDR_ = 0.13) in the striatum remained significant. Regarding specific cognitive domains, the interactions remained significant for Matrix in temporal Centiloids (*β* = − 1.42, *p* = 0.03, *p*_FDR_ = 0.12).

## Discussion

The present study aimed to determine whether regional VR of Aβ PET interacts with *APOE-ε4* status to predict cognitive decline in a cohort of middle-aged CU participants at high risk of AD. Specifically, *APOE-ε4* carriers who were VR+ in lateral temporal regions and in the striatum displayed a significantly steeper cognitive decline, as measured with the PACC, than non-carriers and VR- individuals. This interaction could not be detected with global VR+ and persisted after controlling for hippocampal volume, as a measure of neurodegeneration. The result in the temporal region was replicated when determining regional positivity with quantitative Centiloids-based cut-off values for positivity (Centiloids+). With regard to specific cognitive domains, we also found significant interactions with *APOE-ε4* in temporal regions and the striatum in both VR+ and Centiloids+ on episodic memory and executive function. Again, these associations were not observed with the global measures of amyloid positivity and persisted after controlling for hippocampal volume.

The main novelty of our study resides in that it highlights the value of regional VR of Aβ PET to detect cognitive decline in CU in *APOE-ε4* carriers that, to the best of our knowledge, has not been previously described. These findings are in line with previous reports using regional Aβ positivity using continuous SUVR values [8, 9, 18].

Visual and Centiloid-based determinations of regional Aβ positivity were in high concordance (> 95%), thus showing that regional VR is a valid and comparable alternative to quantitative methods. This is in agreement with our previous assessment of the inter- and intra-reader agreement of regional VRin all brain regions [6]. Taken together, regional patterns of VR positivity, readily available in clinical settings, have a similar capacity as Centiloids-based methods, usually used in research, to assess the risk of cognitive decline associated with regional patterns of Aβ positivity. Therefore, such a good agreement may help bridge methodological discrepancies in regional PET quantification between studies.

According to the spatial–temporal sequencing of Aβ accumulation, the regions with positive Aβ VR that showed a significant interaction with *APOE-ε4* status on cognitive decline (temporal regions and the striatum) are typically considered to be accumulating Aβ later in the AD continuum than those in which this interaction was not found (e.g. frontal and PC/PCC) [7]. Therefore, it is sensible to hypothesize that cognitive decline is closer when Aβ deposition reaches these late-accumulating regions. Supporting this hypothesis, such an interaction was not observed with global measurements of Aβ burden, which are mainly driven by positivity in early regions. In line with our results, recent literature has linked Aβ accumulation in the striatum with a higher risk of cognitive decline among non-demented individuals with elevated cortical Aβ [40].

Our results show that *APOE-ε4* carriers with Aβ VR+ in late amyloid accumulation regions exhibited a significantly steeper cognitive decline compared with non-carriers. This finding is in agreement with previous reports showing that Aβ and *APOE-ε4* interact to influence short-term decline in preclinical AD [3–5]. It is well established that *APOE-ε4* carriers show an earlier and higher cerebral Aβ deposition. On top of this, literature supports the existence of additional mechanisms that may promote cognitive decline in CU *APOE-ε4* carriers with higher Aβ accumulation. Evidence suggests a higher vulnerability of *APOE-ε4* carriers to the toxic effects of Aβ on neuronal integrity that could impact brain processes such as tau phosphorylation, mitochondrial activity, or neuroinflammation [41]. However, many of our results remained significant after controlling for hippocampal volume, suggesting that the effect of neurodegeneration could be regionally specific. Finally, *APOE-ε4* carriers may have higher levels of underlying tau pathology and cerebrovascular disease that could be hypothesized as possible mechanisms underlying cognitive decline in those individuals [41]. In line with this, we found a significant interaction between *APOE-ε4* and VR Aβ accumulation in the striatum with regard to cognitive decline. In agreement with this interpretation, it has been suggested that this association indicates the duration or severity of Aβ burden or signals towards an increase in tau-pathology [14, 40]. Moreover, it has been suggested that tau might partially mediate the deleterious effect of striatal Aβ on cross-sectional cognition [40]. However, the loss of significance in regional Centiloids could be related to quantification in this region which is susceptible of contamination by white matter uptake [6, 7]. Regarding the temporal lobe, a recent study on the sample included here showed that *APOE-ε4* carriers were more prone to Aβ aggregation in temporal areas for any given level of soluble Aβ dyshomeostasis. This finding suggests that *APOE-ε4* facilitates the spread of Aβ in these regions, promoting an earlier co-localization with tau [42] to trigger AD-related neurodegeneration. Taken together, these studies suggest an interplay between patterns of Aβ and tau spread in determining cognitive decline, which would explain why positivity in regions of late Aβ accumulation is associated with cognitive decline in *APOE-ε4* carriers in the present study. Still, previous studies classified the individuals as globally Aβ positive using quantitative methods mostly available in research settings.

The present work mainly measured cognitive change with the PACC, a gold standard global cognitive composite used in the context of the preclinical stage of AD. This measure combines the two cognitive composites for which we have found significant results, namely episodic memory and executive function. In addition, the PACC was constructed to maximize the sensitivity to detect the earliest Aβ-related cognitive changes [11]. Regarding specific cognitive domains, we found an interaction between *APOE-ε4* and Aβ aggregation to promote episodic memory and executive function decline in line with previous reports[18, 19]. We observed that *APOE-ε4* carriers with Aβ VR positivity in the striatum exhibited worse episodic memory performance in the follow-up visit. These results are in alignment with a recent study that found that amyloid accumulation predicts memory decline in 133 CU individuals [8]. In particular, precuneal Aβ burden predicated immediate and delayed episodic memory performance in the whole population, whereas lateral orbitofrontal Aβ burden predicted working and semantic memory performance in Aβ negative baseline group [8]. However, the effect of *APOE-ε4* status in this association was not explored. Here, we found a significant association between decline in episodic memory and Aβ aggregation in a larger and younger sample, although exclusively to *APOE-ε4* carriers and late Aβ accumulation regions. With regard to executive function, we found a significant interaction between *APOE-ε4* and regional Aβ accumulation in temporal and parietal regions and in the striatum. This result is in agreement with the previously mentioned higher risk of cognitive decline with Aβ accumulation in the striatum among non-demented individuals [40]. However, our results demonstrated an additional higher risk for decline in the performance of specific cognitive domains, namely episodic memory and executive function. Therefore, our results show the capacity of regional VR to detect those individuals that have persistent Aβ deposition at the early stages of the AD continuum that, consequently, are at higher risk of cognitive decline.

Some limitations of this work should be considered. First, the number of follow-up visits may be insufficient to interpret results in terms of the temporal evolution of the events. More extended longitudinal studies are required to track the progression of cognitive change related to regional Aβ PET uptake in *APOE-ε4* carriers and non-carriers to improve our understanding of the plausible mechanisms relating the *APOE* genotype and AD pathogenesis. In turn, further longitudinal data collection will help to confirm whether regional VR has a similar prognostic value compared to quantification [6]. Second, replication of the findings presented here in independent cohorts is essential. To overcome these limitations, the second follow-up visit of the longitudinal ALFA+ study is currently ongoing, including both cognition and amyloid PET acquisition, which is being collected in the context of the AMYPAD Consortium [10]. The replication in larger cohorts will also help addressing the relatively few cases of positivity in our study. Nevertheless, we verified that our results were not driven by the presence of a few extreme cases, as no outliers were detected in our sample using standard methods (cases exceeding the median plus or minus 1.5 times the interquartile range). Third, the lack of AD biomarkers at the baseline cognitive assessment is another relevant limitation for interpreting our findings. Even though we cannot know the amyloid status at baseline, evidence shows that Aβ deposition in preclinical AD is slow and protracted, likely to extend for more than two decades [43]. Therefore, given such a slow accumulation rate, 4 years are still a relatively short period to observe major changes in deposited Aβ. Last, in our cohort, individuals with relevant medical conditions or neurologic disease were excluded. As a result, our sample is healthier than expected from an age-matched cohort selected from the general population. Further, participants are younger than those in previous studies [18–20]. However, due to the lack of other comorbidities and the young age in our cohort, the present sample represents the most suitable population for identifying subtle changes in the early stages of the AD continuum, such as cognition.

## Conclusions

In conclusion, we show that *APOE-ε4* carriers with a positive VR Aβ PET in late amyloid-accumulating regions exhibited a significantly worse retrospective cognitive change in the PACC, episodic memory, and executive function. Therefore, our results suggest that late Aβ deposition and *APOE-ε4* carriership combine to determine cognitive decline in CU individuals. In addition, our work supports the value of visual reading in detecting regional Aβ pathology with relevant effects on cognition in individuals at higher risk of developing AD who might benefit from clinical trials or preventive interventions.

## Supplementary Information

**Additional** **file** **1:** **Table** **S1:** Results of linear regression models examining the interaction between number of positive regions and APOE*-*ε4 status on cognitive change. **Figure S1: ** Cross-correlation matrix between regional Centiloids depicting results of Spearman’s correlation.

## Data Availability

Researchers who wish to use data from the ALFA study must obtain approval from the ALFA study management team.

## Abbreviations

AD: Alzheimer’s disease
*A*β: β-Amyloid
CU: Cognitively unimpaired
FDR: False discovery rate
HVa: TIV-adjusted hippocampal volumes
PACC: Preclinical Alzheimer Cognitive Composite
TDFR: Total Delayed Free Recall
TIV: Total intracranial volume
TPR: Total Paired Recall
VR: Visual read
