# Supplementary material for: APOE-ε4 modulates the association between regional amyloid deposition and cognitive performance in cognitively unimpaired middle-aged individuals
Source: EJNMMI Res. 2023 Mar 1;13:18. doi: 10.1186/s13550-023-00967-6 (PMC9978048; doi:10.1186/s13550-023-00967-6)
Supplement: Supplementary file 1 — Additional file 1: Table S1: Results of linear regression models examining the interaction between number of positive regions and APOE-ε4 status on cognitive change. Figure S1: Cross-correlation matrix between regional Centiloids depicting results of Spearman’s correlation. [file 13550_2023_967_MOESM1_ESM.docx]

**Supplementary Information**

***APOE*-ε4 modulates the association between regional amyloid deposition and cognitive performance in cognitively unimpaired middle-aged individuals**

Anna Brugulat-Serrat, Gonzalo Sánchez-Benavides, Raffaele Cacciaglia, Gemma Salvadó, Mahnaz Shekari, Lyduine E. Collij, Christopher Buckley, Bart N.M. van Berckel, Andrés Perissinotti, Aida Niñerola-Baizán, Marta Milà-Alomà, Natàlia Vilor-Tejedor, Grégory Operto, Carles Falcon, Oriol Grau-Rivera, Eider M Arenaza-Urquijo, Carolina Minguillón, Karine Fauria, José Luis Molinuevo, Marc Suárez-Calvet1, Juan Domingo Gispert, for the ALFA Study.

| **Table 1. Results of linear regression models examining the interaction between number of positive regions and APOE-ε4 status on cognitive change** | | | | | | | | | | | | | | | | | | | | |
| --- | --- | --- | --- | --- | --- | --- | --- | --- | --- | --- | --- | --- | --- | --- | --- | --- | --- | --- | --- | --- |
|  |  | **PACC** | | |  | **TPR** | | |  | **Matrix** | | |  | **DS Backward** | | |  | **Coding** | | |
|  |  | **β (SE)** | ***p*** | ***p*_FDR_** |  | **β (SE)** | ***p*** | ***p*_FDR_** |  | **β (SE)** | ***p*** | ***p*_FDR_** |  | **β (SE)** | ***p*** | ***p*_FDR_** |  | **β (SE)** | ***p*** | ***p*_FDR_** |
| ***APOE*-ε4 status x Number of positive VR regions** | | | | | | | | | | | | | | | | | | | | |
| VR |  | -0.15 (0.09) | 0.09 | 0.16 |  | -0.13 (0.08) | 0.13 | 0.15 |  | -0.19 (0.08) | 0.02* | 0.03* |  | -0.10 (0.08) | 0.21 | 0.33 |  | -0.14 (0.09) | 0.12 | 0.16 |
| CL |  | -0.10 (0.09) | 0.24 | 0.32 |  | -0.08 (0.08) | 0.36 | 0.41 |  | -0.14 (0.08) | 0.09 | 0.15 |  | -0.11 (0.08) | 0.19 | 0.30 |  | -0.14 (0.09) | 0.13 | 0.17 |
| Model: Cognitive change= Number of positive regions x *APOE-ε4* status + regional CL or VR + *APOE-ε4* status + age + sex + education + cognitive baseline score.  *CL* Centiloids, *DS* digit span, *PACC* Preclinical Alzheimer Cognitive, *TPR* total paired Recall, *VR* visual read  **p*<0.05 | | | | | | | | | | | | | | | | | | | | |


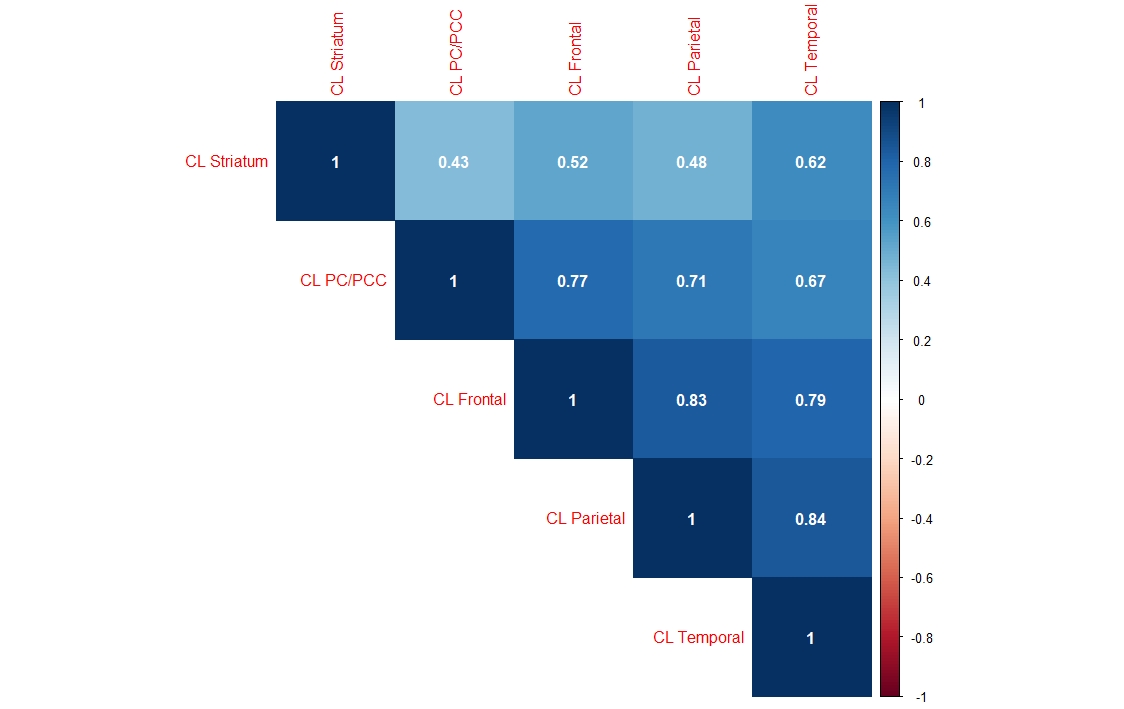


**Figure 1.** Cross-correlation matrix between regional Centiloids depicting results of Spearman’s correlation. All correlations had a p-value <0.001.
